# Sporadic intra-abdominal desmoid tumor with a very unusual onset: two case reports

**DOI:** 10.1186/s13256-021-03058-z

**Published:** 2021-09-16

**Authors:** Hiroshi Kuwabara, Sou Katayanagi, Itsuki Koganezawa, Masashi Nakagawa, Kenji Katsumata, Akihiko Tsuchida, Shigeyuki Kawachi

**Affiliations:** 1 Department of Digestive and Transplantation Surgery, Tokyo Medical University Hachioji Medical Center, 1163 Tatemachi, Hachioji, Tokyo 193-0998 Japan; 2 Department of Gastrointestinal and Pediatric Surgery, Tokyo Medical University, 6-7-1 Nishishinjuku, Shinjuku Ward, Tokyo, 160-0012 Japan

**Keywords:** Aggressive fibromatosis, Mesenteric fibromatosis, Surgery, Tumoral hemorrhage, Obstructive uropathy

## Abstract

**Background:**

Intra-abdominal desmoid tumors are rare soft tissue tumors that arise mainly in the mesentery and pelvis. Their etiology may include genetic mutations, estrogen-associated changes after childbirth, and mechanical factors such as a history of abdominal surgery. However, there are cases of intra-abdominal desmoid tumors that develop in the absence of such causes. Since they are rare, diagnosis is often difficult based on clinical findings. We encountered two cases of patients with sporadic intra-abdominal desmoid tumors with a very unusual onset and contrasting features.

**Case presentation:**

The first patient was a 51-year-old asian man who presented with sudden onset of abdominal pain. He was referred to our department because of a giant tumor detected on abdominal ultrasonography. Imaging revealed a 19-cm tumor with internal tumoral hemorrhage; however, no definitive diagnosis was made. Tumor resection was performed for diagnostic and therapeutic purposes. The second patient was a 41-year-old asian man, and right hydronephrosis was detected on abdominal ultrasonography during a periodic medical checkup. We diagnosed invasion of the primary mesenteric tumor into the right ureter using diagnostic imaging and performed ileocecal resection with partial right ureteral resection for a definitive diagnosis and therapeutic purposes. Although the tumors of both patients had developed from the ileal mesentery, the tumors were substantially different from each other based on their imaging findings, macroscopic morphology, and progression pattern. Meanwhile, they showed similar pathological characteristics. Both consisted of bundles of collagen fibrils of spindle-shaped fibroblasts with low cell atypia. Moreover, they were diagnosed as desmoid tumors using positive immunohistochemical staining for β-catenin.

**Conclusions:**

Neither patient had susceptibility factors for desmoid tumors, and to our knowledge, there have been very few reports to date of intra-abdominal desmoid tumors that were diagnosed because of acute abdominal pain caused by tumoral hemorrhage or asymptomatic obstructive uropathy. Furthermore, it is clinically interesting that the two patients showed contrasting progression patterns and imaging findings. Intra-abdominal desmoid tumors are rare and may present with various symptoms and findings similar to those observed in our patients. Diagnosis therefore requires experience and knowledge that is not bound by preconceptions.

## Background

Desmoid tumors, whose name is derived from the Greek *desmos*, which means “band- or tendon-like,” are stromal tumors that develop from fibrous connective tissue and were first described in 1838 [1]. The National Comprehensive Cancer Network (NCCN) guidelines describe this type of tumor as an aggressive fibromatosis, which does not metastasize but grows locally aggressive and has a very high local recurrence rate even after complete resection [2]. Their etiology is thought to be multifactorial. Moreover, it is widely known that germline mutations in the adenomatous polyposis coli (*APC*) gene, which cause familial adenomatous polyposis (FAP), are associated with the development of desmoid tumors, and it has been reported that the risk of onset of desmoid tumors in FAP patients is 800 times that in a healthy person [3]. In addition to the *APC* gene, somatic mutations of the catenin beta-1 (*CTNNB1*) gene are involved in the development of sporadic desmoid tumors, and it has been reported that *CTNNB1* gene mutations are found in approximately 50–85% of cases [4–6]. Both genes are involved in important signal transduction pathways that play roles in regulating the cellular level of β-catenin, which acts on cell–cell junctions. Therefore, when a mutation occurs in either *APC* or *CTNNB1*, β-catenin accumulates in cells, forming tumors [7]. It has also been reported that estrogen is involved in the development and growth of desmoid tumors; hence, they often occur in women, particularly in multiparous women. Estrogen receptors are highly expressed in a large number of desmoid tumors, and tamoxifen has recently been reported to be effective as a treatment [8, 9]. A history of trauma or surgery is also considered to be a factor in the development of desmoid tumors, and mutations in genes involved in the wound healing process of tissues are considered to be a cause of desmoid tumor development [10]. Intra-abdominal desmoid tumors are often found mainly as abdominal distensions or bowel obstructions associated with tumor growth or invasion [11, 12]. It is often difficult to differentiate them from other mesenchymal tumors such as gastrointestinal stromal tumors (GISTs) and sarcomas by diagnostic imaging. Here, we report two cases of intra-abdominal desmoid tumors without any causative factors. Sporadic intra-abdominal desmoid tumors are relatively rare, and both of our cases had an unusual onset. We believe that these two cases of desmoid tumors, which showed contrasting characteristics in terms of imaging findings and progression patterns, are useful for diagnostic and therapeutic strategies in clinical practice.

## Case presentation

**Fig. 1:**
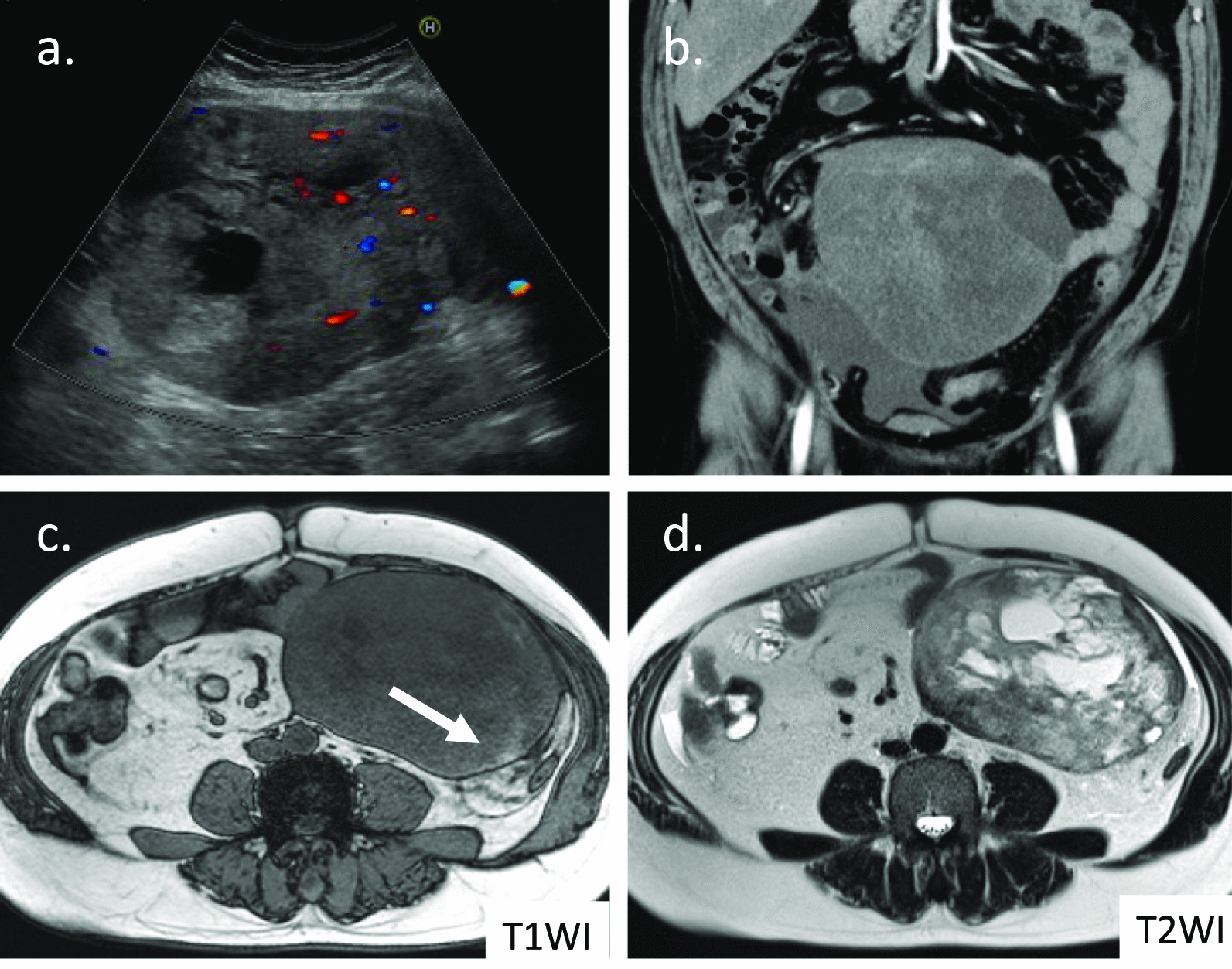
Diagnostic findings in Case 1.** a** Abdominal ultrasonography displaying a large solid mass with cystic degeneration, and an irregular echoic pattern with vascular signals inside. **b** Abdominal contrast-enhanced computed tomography displaying a large, well-defined circular tumor with a heterogeneous interior, which appears to be partially continuous with the small intestine. **c** Magnetic resonance imaging displaying some high-intensity signals (white arrow) on T1-weighted image. **d** Cyst components are scattered on T2-weighted image

**Fig. 2:**
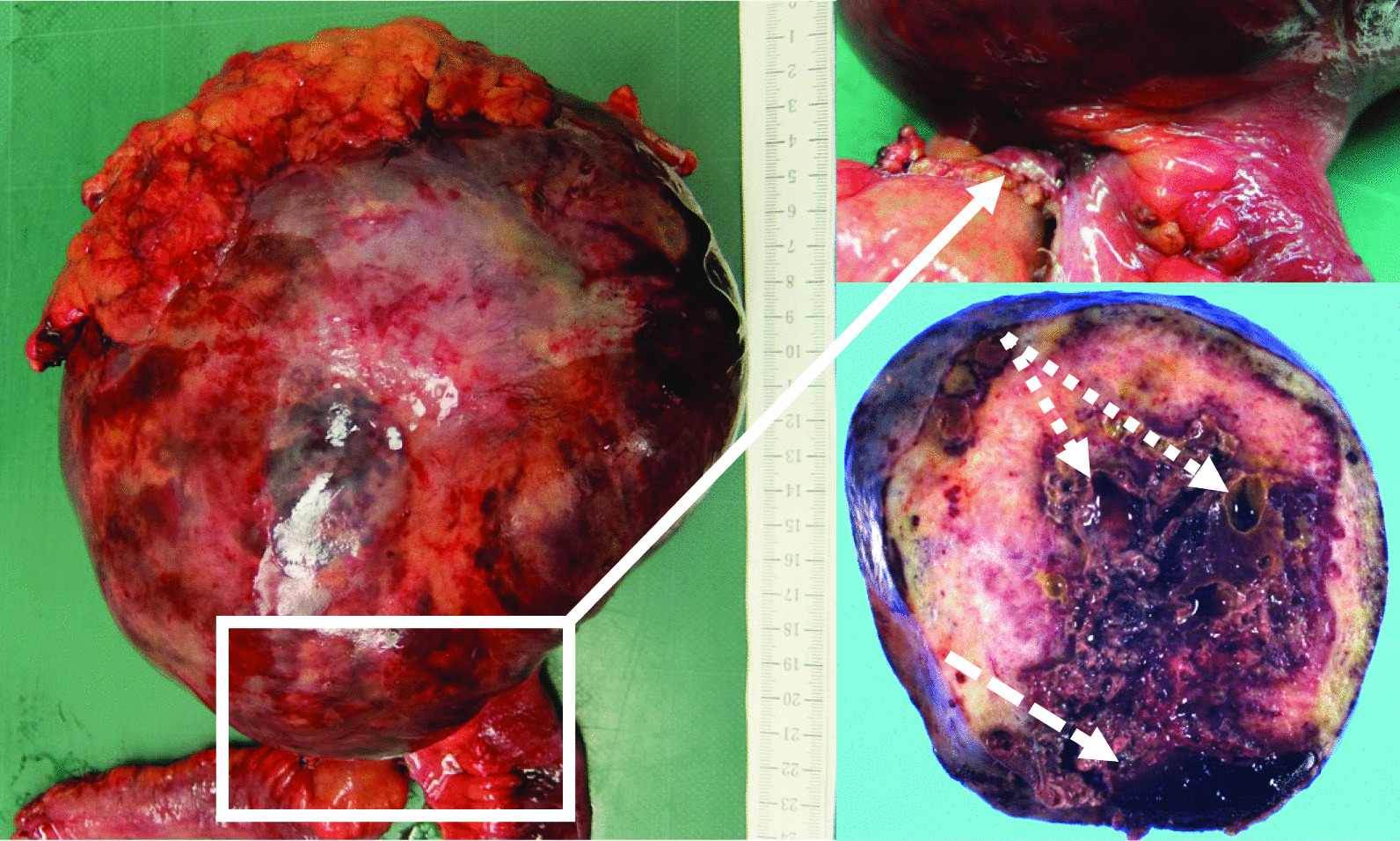
Macroscopic characteristics of the tumor in Case 1. The resected specimen appears to originate from the small mesentery (white arrow). The tumor is grossly covered by a capsule, and the surface is smooth, elastic, and soft. The split surface contains hematoma (white dashed line) and cyst components (white dotted arrow)

**Fig. 3:**
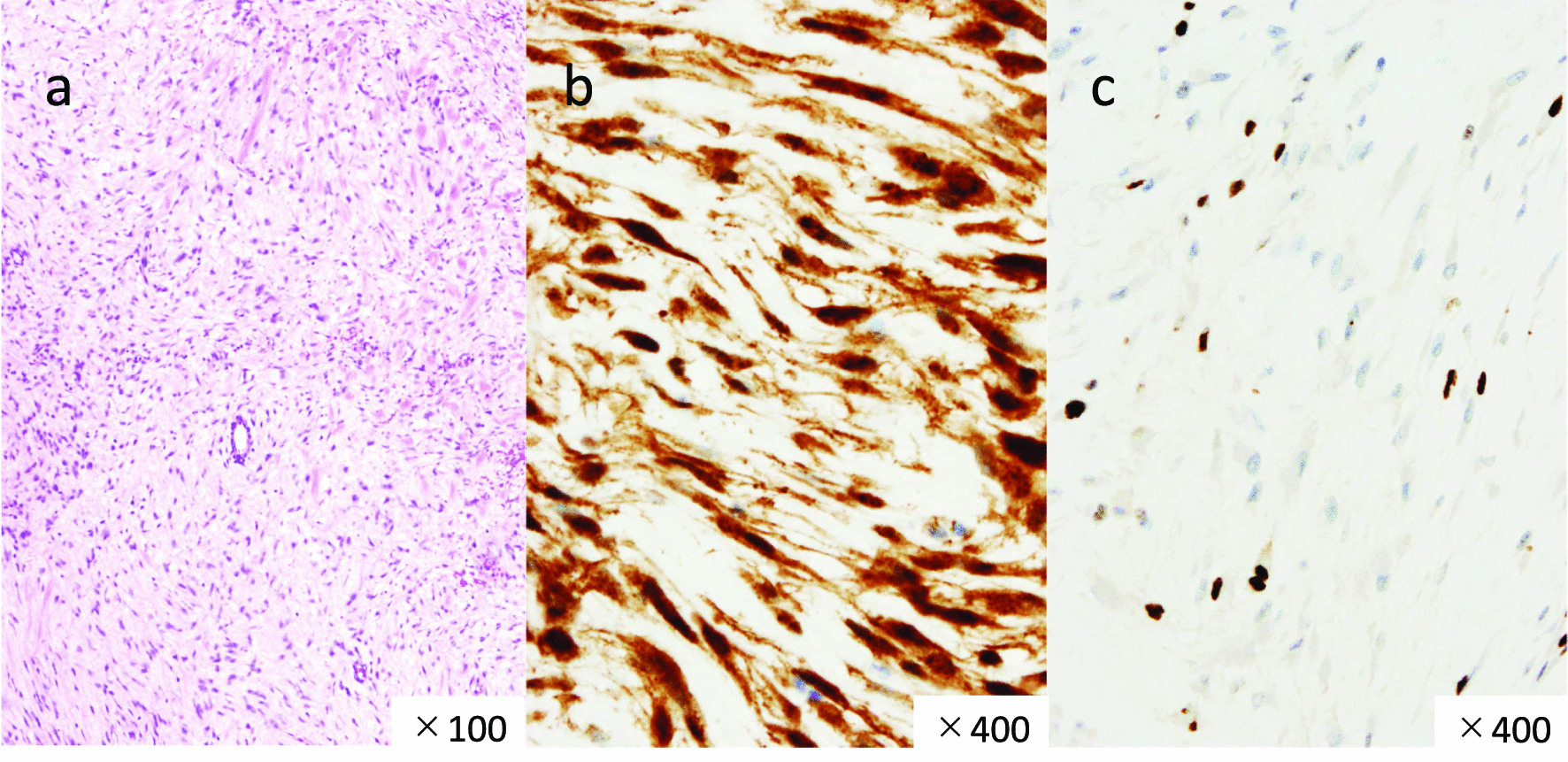
Histological and immunohistological features of desmoid tumors in Case 1. **a** Hematoxylin and eosin (HE) staining demonstrating the proliferation of low-density stromal cells forming thick collagen fibrils (low-power view). **b** Strong immunostaining for β-catenin (high-power view). **c** Expression of Ki-67 analyzed by immunostaining. The labeling index was 6% (high-power view)

Case 1 involved a 51-year-old asian man who presented to the clinic with sudden left abdominal pain. Ultrasonography revealed a smooth and solid intra-abdominal mass with a maximum diameter of approximately 19 cm, which contained cystic components (Fig. 1a). He was referred to our hospital for further examination. He had been healthy and had no history of hospitalization or abdominal surgery. A tender and mobile infant-head-sized mass in the left abdomen was detected by palpation. On enhanced abdominal computed tomography (CT), the tumor appeared as a well-circumscribed mass with scattered density, which appeared to arise from the small intestine (Fig. 1b). Abdominal magnetic resonance imaging (MRI) revealed a region with a high signal on T1-weighted images (T1WI) in a segment of the tumor, suggesting intratumoral hemorrhage (Fig. 1c). Cyst components were displayed on T2-weighted imaging (T2WI) (Fig. 1d). Small intestine endoscopy demonstrated ischemic mucosal changes 20 cm from the ileocecal valve, and gastrointestinal imaging displayed slight stenosis due to exclusion from the external intestinal wall. Based on the above findings, GIST was first considered in the differential diagnosis, and surgery was performed for therapeutic diagnosis. The tumor was in the mesentery and it involved a part of the proximal ileum, 20 cm from the terminal ileum. Therefore, a small intestinal resection was performed. The tumor measured 19 × 16.5 × 9 cm with a capsule that originated from the ileum mesentery. Moreover, cystic components and hematoma were observed on the split surface (Fig. 2). Histopathological findings showed that the proliferation of bland spindle-shaped cells with minimal mitotic activity caused the formation of a broad bundle of stromal fibrosis (Fig. 3a). The tumor was diagnosed as a desmoid tumor based on positive immunohistochemical staining for β-catenin (Fig. 3b) and negative staining for c-kit, CD34, platelet-derived growth factor (PDGFRα), and Discovered on GIST-1 (DOG-1). The Ki-67 labeling index, which is an index of cell proliferation, was approximately 6% (Fig. 3c). The surgical margin was pathologically negative, indicating complete resection of the desmoid tumor. The patient is still being followed up with CT imaging every 6 months postoperatively, and has survived for 3 years without any recurrence.

**Fig. 4:**
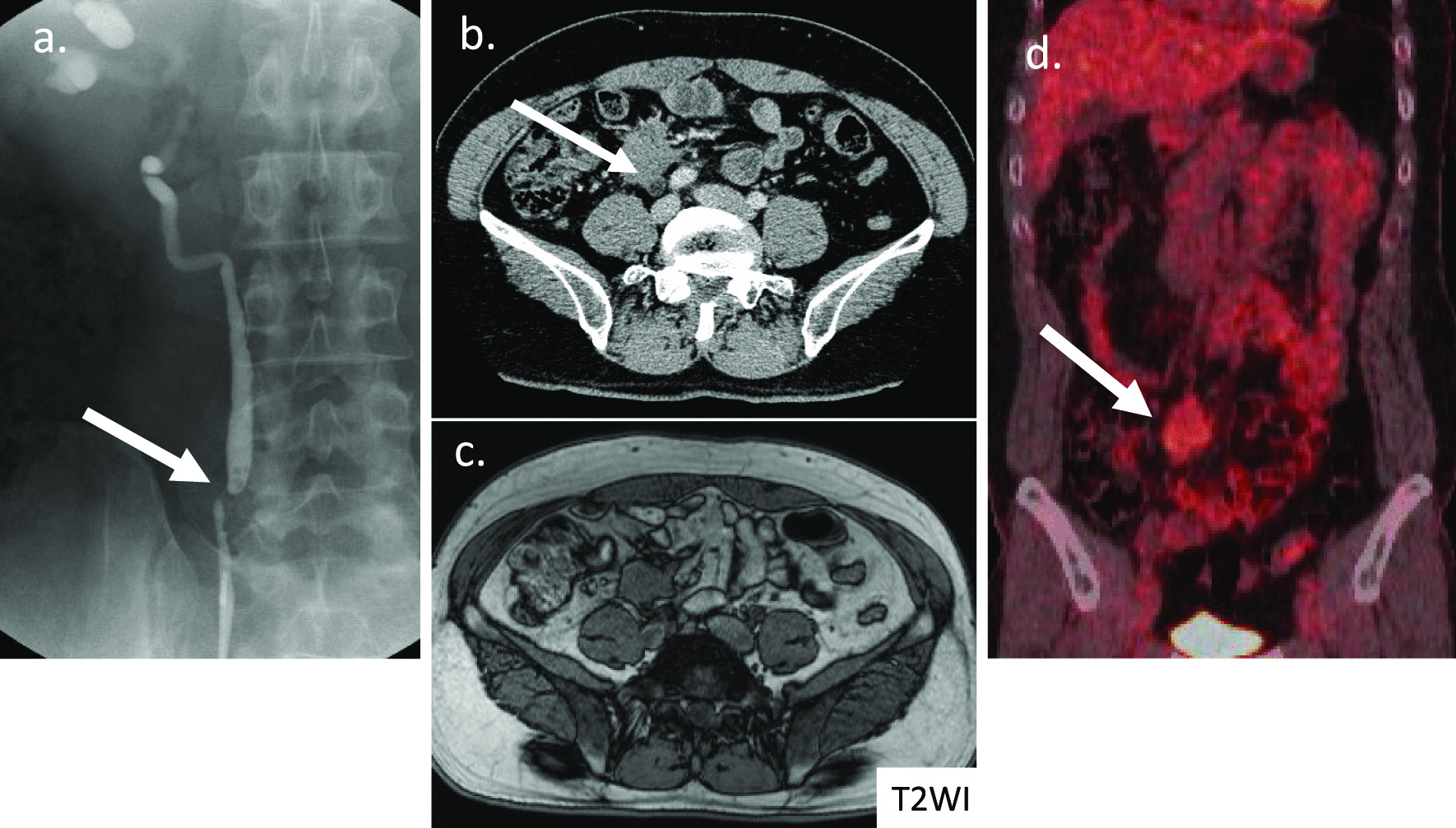
Diagnostic findings in Case 2.** a** Drip infusion pyelography displaying right uropathy stenosis (white arrow). **b** Abdominal contrast-enhanced computed tomography (CT) displaying a homogeneously enhanced 35-mm mass with an irregular, spiculated margin encasing the right dilated ureter at the level of the right common iliac artery (white arrow). **c** T2-weighted image displaying a low signal mainly at the center of the tumor. **d** ^18^F-Fluorodeoxyglucose positron emission tomography (FDG-PET) CT displaying slightly increased FDG in the mass (white arrow)

**Fig. 5:**
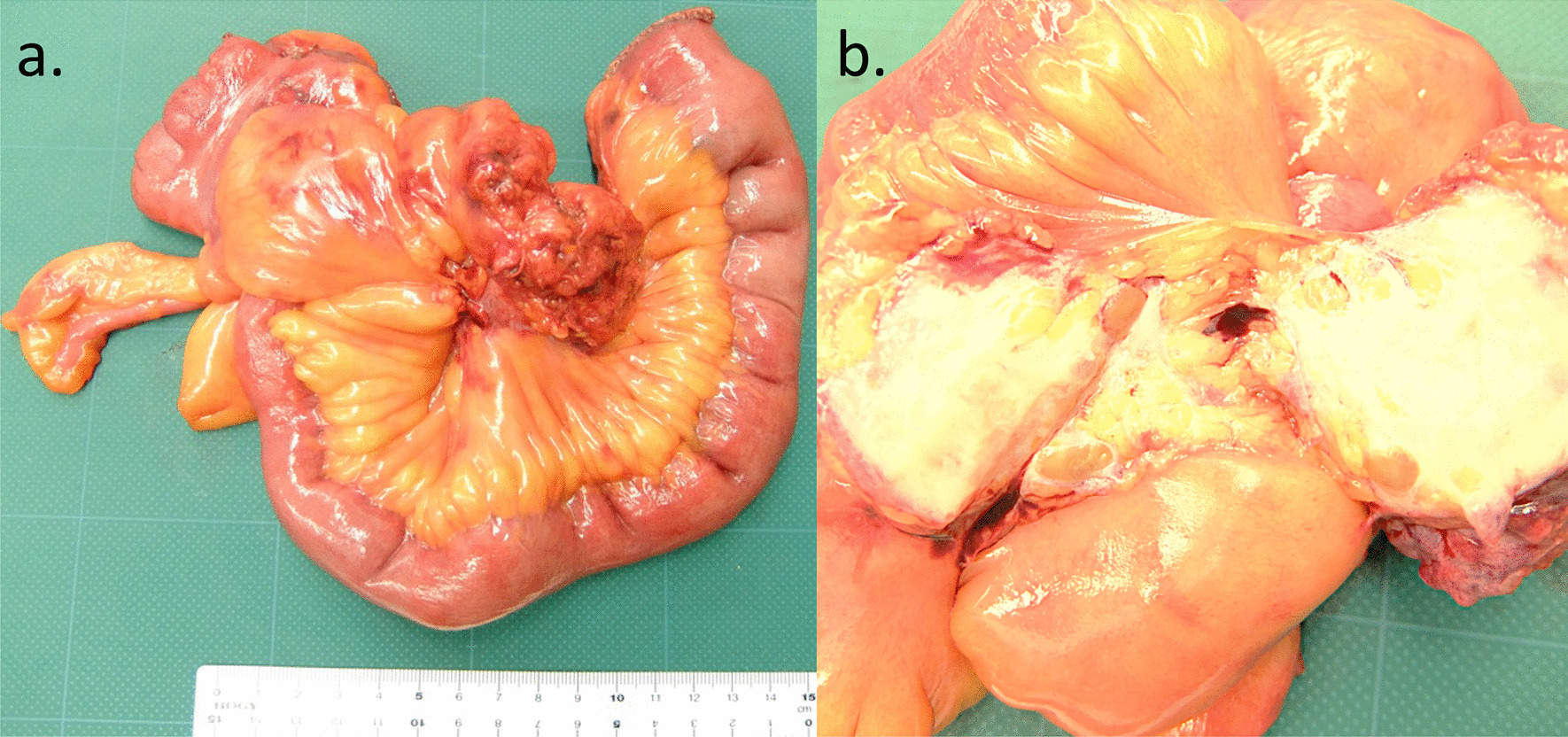
Macroscopic findings of the resected specimen in Case 2.** a** A bulky mass with tentacle-like spiculated extensions with infiltrative growth is located at the center of ileum. **b** The cut surface of the tumor is equally whitish, fibrous, and firm, partially involving the mesenteric adipose tissues

**Fig. 6:**
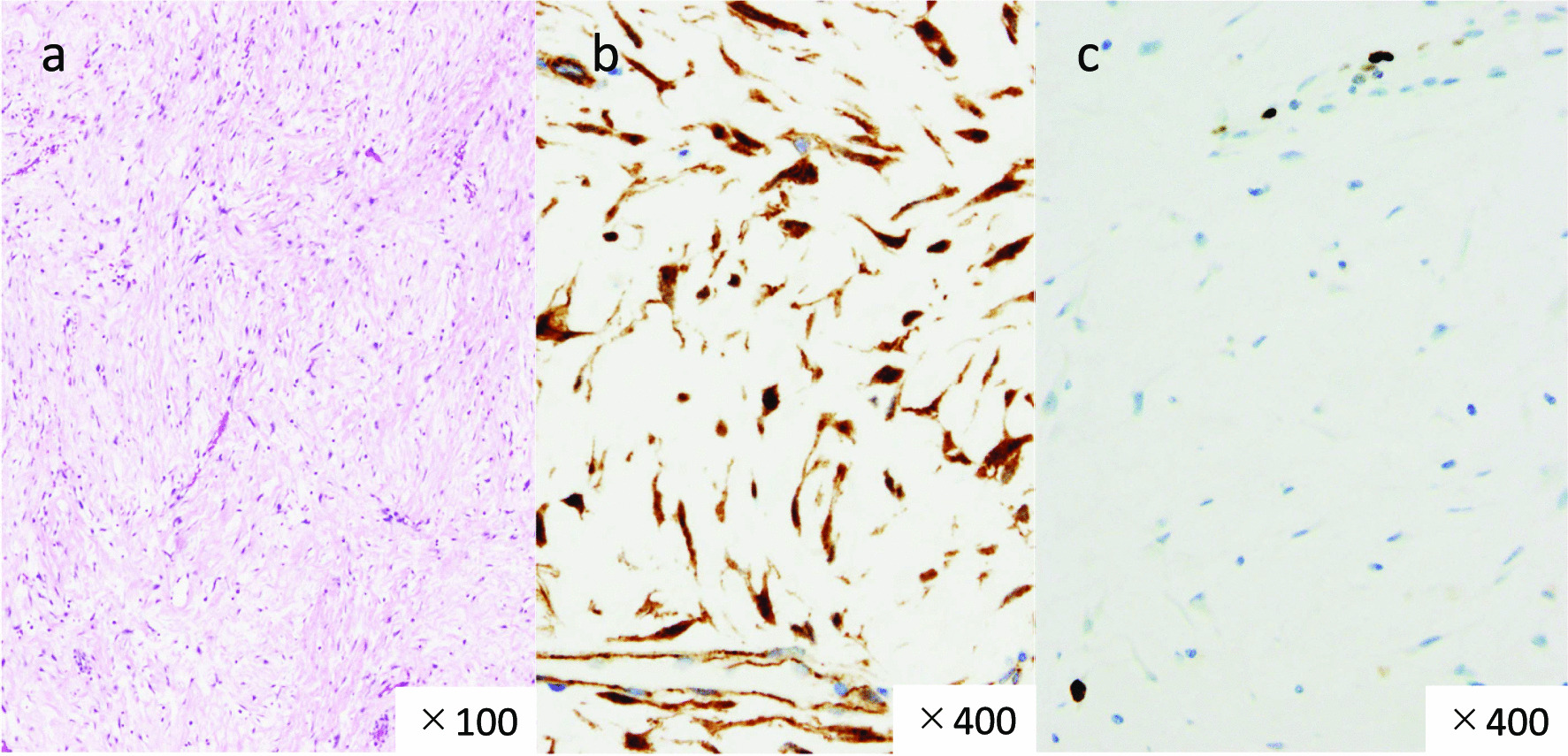
Histological and immunohistological features of desmoid tumors in Case 2. **a** Hematoxylin and eosin (HE) staining shows proliferation of spindle-shaped cells. Wavy collagenous bands of fiber lie in stroma (low-power view). **b** Immunohistological staining for β-catenin shows strongly positive staining of cell (high-power view). **c** Immunohistological expression of Ki67 in tumor. Labeling index =1% (high-power view)

Case 2 involved a 41-year-old asian man who had no subjective symptoms and was diagnosed with right hydronephrosis on abdominal ultrasonography during a periodic health checkup. He was referred to our hospital for urological examination. He had no history of abdominal trauma, surgery, or genetic disease. Physical examination, serum biochemistry analysis, and upper and lower gastrointestinal endoscopy did not reveal any abnormal findings. However, drip infusion pyelography revealed narrowing of the right ureter (Fig. 4a), and abdominal pelvic CT revealed a 3.5-cm tumor that had uniform contrast with the dilated ureter (Fig. 4b). MRI T2WI showed a low signal mainly at the center of the tumor (Fig. 4c). On ^18^F-fluorodeoxyglucose positron emission tomography (FDG-PET), mild accumulation of FDG in the late phase was observed at the site of the tumor (Fig. 4d). This slight increase in the overall accumulation was proven by the change in the maximum standardized uptake value in the early (3.50 seconds) to late (4.51 seconds) phase. Based on the diagnostic findings, the tumor was suspected of being a desmoid tumor, since it was a low-grade but strongly invasive tumor. Surgery was performed to establish a definite diagnosis and treatment for the patient. The 4-cm hard mass located at the ileum mesentery had grown and infiltrated its surrounding tissue. Because the dorsal side of the tumor was invading the ureter and hence could not be dissected, ileocecal resection along with a portion of the right ureter (5 cm in the major axis) was performed. Histological analysis showed that the 3.7 × 3.5 × 3.2-cm tumor (Fig. 5) had invaded the adventitia of the ureter. Based on pathological findings, the tumor comprised infiltrating and proliferating spindle-shaped fibroblasts with low cell atypia, characterized by a keloid-like eosinophilic collagen fiber bundle (Fig. 6a). Immunostaining demonstrated that the tumor cells were negative for c-kit, CD34, PDGFRα, and DOG-1. However, a strong positive result for β-catenin was observed in the cytoplasm and nucleus (Fig. 6b). These findings led to the diagnosis of a desmoid tumor. The Ki-67 labeling index was less than 1% (Fig. 6c). The lesion was completely excised with clear margins. The patient is still being followed up with CT imaging every 6 months postoperatively and has survived for 3 years without any recurrence (Table 1)

**Table 1:** Desmoid tumor characteristics in Cases 1 and 2

	Case 1	Case 2
Gender/age	Male/55	Male/41
Past medical history and family history	Not applicable	Not applicable
Symptoms	Acute abdominal pain	Asymptomatic
Imaging features	Very large size of heterogeneous and well-defined circular tumor with cystic components	Small size of homogenous tumor with irregular and spiculated margin
Surgical features	Elastic and expansive giant tumor on small mesentery	Small and extremely invasive solid tumor on small mesentery
Pathological features (immunohistochemical staining)	β-catenin: strongly positive, c-kit, CD34, PDGFRα, and DOG-1: negative Ki-67 labeling index = 6%	β-catenin: strongly positive, c-kit, CD34, PDGFRα, and DOG-1: negative Ki67 Labeling index =1%

## Discussion and conclusions

Desmoid tumors account for 0.03% of all neoplasms and less than 3% of soft tissue tumors; hence, it is a very rare disease. Approximately 10–20% of all desmoid tumors occur in the abdominal cavity [13–15], most of which occur due to FAP, pregnancy and childbirth, or history of abdominal surgery. Therefore, sporadic intra-abdominal desmoid tumors, such as ours, are extremely rare.

In our first patient, sudden left abdominal pain led us to the diagnosis. MRI revealed an intratumoral hemorrhage, suggesting that the abdominal pain was due to the rapid tumor capsule extension. To date, there has been only one reported case of a desmoid tumor that was discovered due to intratumoral hemorrhage [16]. In our second patient, right hydronephrosis was detected using abdominal ultrasonography. There are relatively many reports of desmoid tumors that cause ureteral obstruction. However, most of these are found in advanced cases at the time of diagnosis [17]. As seen in our second patient, the tumor arose near the right ureter on the proximal side of the ileum mesentery. This caused the right hydronephrosis when the tumor was still small.

Intra-abdominal desmoid tumor is a non-organ-specific mesenchymal tumor that can occur anywhere in the abdominal cavity. Therefore, it is necessary to differentiate it from GIST, sarcoma, lymphoma, neurogenic tumor, and so on. However, preoperative histological diagnosis is often difficult because, unlike cancer, desmoid tumors do not originate from the epithelium, making it difficult to perform biopsies using endoscopes. Needle biopsy is sometimes used, but desmoid tumors consisting of fibrous connective tissue are often difficult to collect in sufficient quantities [12]. In addition, it is not useful because of the difficulty and the possibility of dissemination. In our cases, we did not perform needle biopsy because we suspected GIST in our first case and were concerned about the possibility of dissemination. Moreover, it was difficult to perform the procedure in our second case.

Regarding the diagnostic imaging of desmoid tumors, Huss *et al*. reported that the preoperative accuracy of detecting intra-abdominal desmoid was approximately 40%, and that it was difficult to discriminate it from GISTs. The differences between desmoid tumors and GISTs in diagnostic imaging include their site of occurrence, shape, contrast effects, and tumor content. GISTs originate from the gastrointestinal tract and are characterized mainly as well-defined circular or lobulated tumors with a heterogeneous contrast effect, sometimes with necrotic vessels and cysts inside. On the other hand, desmoid tumors, originating from outside the gastrointestinal tract, often have a morphologically invasive form, relatively uniform contrast effects, and rarely have necrosis or cysts inside [18]. In the imaging analyses of our first patient, the tumor had a well-defined circular shape. The inside of the tumor was heterogeneous on imaging, accompanied by necrosis and cyst formation, which were very different from the previously reported imaging characteristics of desmoid tumors and were characteristic findings of GISTs. In contrast, in our second patient, the mesenteric tumor had a uniform contrast effect, an irregular rim shape, and invasive growth, which was consistent with the characteristics of desmoid tumors. Hauli *et al*. reported the correlation between CT features and the Ki-67 index in GISTs [19]. According to their report, the mean tumor size was significantly greater in the group with Ki-67 > 5% than in the group with Ki-67 ≤ 5%, and features of necrosis or cystic degeneration were often observed in the group with Ki-67 index > 5%. It seems that the same reasoning can be applied to the desmoid tumor in our first patient. In the first patient, the Ki-67 index was 6%, which is much higher than the 1% in the second patient. That is, in the first patient, it can be inferred that necrosis inside the tumor occurred due to rapid tumor growth. In our second patient, FDG-PET was performed to rule out other primary lesions. It also displayed slight FDG accumulation in the main lesion, indicating no sign of malignancy. FDG accumulation is generally low in desmoid tumors [20] because it reflects tumor burden and cell proliferation, and hence is useful in differentiating different types of malignancies. Based on the above findings, our first patient was initially diagnosed with GIST, while our second patient was suspected of having a desmoid tumor.

The treatment options for desmoid tumors are diverse. We performed surgery in both patients since it was determined that surgical resection was necessary and possible. In our first patient, we diagnosed GIST preoperatively, so we tried to perform complete resection and protective operation to avoid the possibility of recurrence and dissemination. In our second patient, we planned a combined ureteral resection preoperatively, assuming that it would be difficult to resect the tumor from the ureter. The NCCN guidelines suggest that careful observation of histologically confirmed desmoid tumors is a treatment option only for slow-growing cases without functional restriction. Fiore *et al*. reported that half of the cases of “wait and see” were free of recurrence for 5 years [21] However, there are no precise treatment guidelines specific to intra-abdominal desmoid tumors. Surgery may be the first choice for patients with rapidly growing tumors or those at risk of losing organ function due to tumor invasion of other organs. As in our second patient, intra-abdominal desmoids with obstructive uropathy often require ureteral resection. If desmoid tumors are suspected before surgery, a treatment strategy that includes ureteral resection is necessary [22]. Additionally, the NCCN guidelines state that if surgical resection significantly impairs function or if the tumor is difficult to resect, nonsteroidal anti-inflammatory drugs (NSAIDs), hormonal therapy, and systemic therapy based mainly on chemotherapy and molecular-targeted agents should be used. Radiation therapy may also be indicated if remnants are suspected after resection. However, radiation therapy for intra-abdominal desmoids carries the risk of radiation enteritis, and hence is not recommended by the NCCN guidelines. Even though complete surgical resection was achieved in our patients, the above-stated systemic therapies should have been considered if macroscopic residuals were suspected. However, it should be noted that treatments other than surgical resection for desmoid tumors have not been established and are still in the experimental stage.

Macroscopically, the appearance of the tumors was completely different between our two patients. In our first patient, the tumor grew expansively and showed necrosis, cysts, and hematoma, which are rare in desmoid tumors. In contrast, in our second patient, the tumor was hard like a stone and showed highly invasive growth. On the other hand, histologically, both tumors showed similar characteristics. Desmoid tumors are characterized by the ordered arrangement of spindle-shaped fibroblasts with low cell atypia and mitosis, and more abundant collagen fibrils than GISTs, which are also mesenchymal tumors [23]. Positive immunostaining for β-catenin contributed in the diagnosis of desmoid tumors [24], and negative immunostaining for c-kit, CD34, PDGFRα, and DOG-1 contributed in clearly distinguishing them from GISTs. The tumors from our patients had all of the mentioned characteristics. Ki-67, an indicator included in the diagnostic criteria, is a risk factor for malignancy and recurrence in GISTs as well as other neoplastic diseases, which suggests that the tumor in our first patient had higher malignancy than that in our second patient. However, Ki-67 is not well documented as a prognostic or risk factor for recurrence in desmoid tumors [25, 26]. This is thought to be because desmoid tumors do not have metastatic potential, even when cell proliferation is rapid. Complete resection without pathological remnants is considered important to achieve a low risk of recurrence and a favorable prognosis [27, 28]. Thus, complete resection was performed in both patients.

There is still no clear guideline for postoperative surveillance of desmoid tumors. Regarding the recurrence of desmoid tumors, it has been reported that the number of patients with FAP is significantly higher than that of patients with sporadic desmoids. It was also found that more than 80% of them occurred in the intra-abdominal cavity after colon surgery. This is because the tumor diameter tends to be large in FAP-related recurrence, making complete resection difficult to achieve. He *et al*. reported that incomplete resection (marginal status R1 or close to R1), large tumor diameters, tumors in the extremities, and younger age at onset are risk factors for recurrence [28]. In our first patient, the tumor size was very large, which is considered a risk factor for recurrence. Currently, we are conducting surveillance using CT images every 6 months. Even if the desmoid tumors were completely resected, the 5-year recurrence-free survival rate is very low (14), so careful follow-up will be necessary in the future.

We encountered two patients with sporadic intra-abdominal desmoid tumors with unusual onset. Intra-abdominal desmoid tumors can cause various symptoms and conditions depending on the site of occurrence. Moreover, radiographic study findings are also diverse, which makes diagnosis difficult. Therefore, diagnosis may require knowledge and experience that is not limited by preconceptions. In our patients, complete resection was possible. However, the local recurrence rate is extremely high, so careful follow-up is required.

## Data Availability

Not applicable.

## Abbreviations

GIST: Gastrointestinal stromal tumors
CT: Computed tomography
NCCN: National Comprehensive Cancer Network
MRI: Magnetic resonance imaging
FAP: Familial adenomatous polyposis
T1WI: T1-weighted image
FDG-PET: ^18^F-Fluorodeoxyglucose positron emission tomography
APC: Adenomatous polyposis coli
HE: Hematoxylin and eosin
